# Wnt and RUNX2 mediate cartilage breakdown by osteoarthritis synovial fibroblast‐derived ADAMTS‐7 and ‐12

**DOI:** 10.1111/jcmm.14283

**Published:** 2019-03-22

**Authors:** Selene Pérez‐García, Mar Carrión, Raúl Villanueva‐Romero, Tamara Hermida‐Gómez, Mercedes Fernández‐Moreno, Mario Mellado, Francisco J. Blanco, Yasmina Juarranz, Rosa P. Gomariz

**Affiliations:** ^1^ Departamento de Biología Celular, Facultad de Biología Universidad Complutense de Madrid Spain; ^2^ Servicio de Reumatología, Instituto de Investigación Biomédica de A Coruña (INIBIC), Complexo Hospitalario Universitario de A Coruña, Sergas Universidade de A Coruña (UDC) A Coruña Spain; ^3^ Departamento de Inmunología y Oncología Centro Nacional de Biotecnología (CNB)/CSIC Madrid Spain

**Keywords:** ADAMTS‐12, ADAMTS‐7, COMP, extracellular matrix, osteoarthritis, Runx2, synovial fibroblasts, Wnt signalling

## Abstract

Failure of therapeutic approaches for the treatment of osteoarthritis (OA) based on the inhibition of metalloproteinases, might be because of their constitutive expression in homeostasis, together with their network complexity. The knowledge of this network would contribute to selective target pathological conditions. In this sense, blockade of mediators produced by neighbouring joint cells, such as synovial fibroblasts (SF), would prevent cartilage damage. Thus, we studied the contribution of ADAMTS‐7 and ‐12 from SF to cartilage oligomeric matrix protein (COMP) degradation, and the signalling pathways involved in their expression. We report for the first time in SF, the involvement of ERK‐Runx2 axis and Wnt/β‐catenin signalling in ADAMTS‐12 and ADAMTS‐7 expressions, respectively, with the subsequent consequences in COMP degradation from cartilage extracellular matrix. After stimulation with IL‐1β or fibronectin fragments, we showed that ERK inhibition decreased Runx2 activation and ADAMTS‐12 expression in OA‐SF, also reducing Fn‐fs‐induced COMP degradation. Blockage of Wnt signalling by DKK1 reduced ADAMTS‐7 and COMP degradation in OA‐SF as well. In addition, Wnt7B expression was induced by IL‐1β and by itself, also increasing ADAMTS‐7. Our results could contribute to the development of disease‐modifying OA drugs targeting ADAMTS‐7 and ‐12 for the prevention of extracellular matrix components degradation like COMP.

## 1 INTRODUCTION

Osteoarthritis (OA) is the main cause of incapacity in elderly population, although other factors such as obesity, metabolism and previous joint damage also generate patients’ disability. OA is characterized by loss of cartilage extracellular matrix (ECM) homeostasis, leading to its destruction. Despite cartilage degradation is the most remarkable symptom of OA, other tissues of the joint are also affected, including subchondral bone, tendons, ligaments as well as the synovial membrane. Matrix‐degrading enzymes released by chondrocytes and synovial fibroblasts (SF) induce cartilage matrix degradation in OA.^1^ It is well known the role of zinc‐dependent proteases in the osteoarthritic degradation of the ECM, including matrix metalloproteinases (MMPs) and several ADAMTS (a disintegrin and metalloproteinase with thrombospondin motifs), such as the aggrecanases ADAMTS‐4 and ‐5.^2^, ^3^ Given the limited regeneration capacity of the articular cartilage, prevention of its damage, results crucial to reduce disease severity. In this sense, the control of inflammation and proliferation of the synovial membrane in OA patients could contribute to cartilage protection.

In rheumatoid arthritis (RA) SF are main players involved in joint destruction, but little is known about their contribution to the pathology of OA. It is known that SF release mediators that contribute to joint destruction and to the chronicity of inflammation. Although metalloproteinases have been identified as targets of therapeutic intervention, they probably failed because of their role in homeostasis, remodelling and wound healing or might be because of the diversity of the metalloproteinases network and the complexity of their interrelations.^4^ The knowledge of the location and function of this intricate network could contribute to the design of selective targets to prevent cartilage destruction.

We have recently reported the involvement of SF in the production of ADAMTS‐4, ‐5, ‐7 and ‐12, and their contribution to ECM damage.^5^ The participation of aggrecanases in cartilage destruction is well known, but the specific contribution of ADAMTS‐7 and ‐12 is less established. ADAMTS‐7 and ‐12 digest cartilage oligomeric matrix protein (COMP),^6^, ^7^, ^8^ a non‐collagenous component of cartilage. COMP is a protein of the ECM that contributes to the maintenance of cartilage stability through its interaction with other components such as aggrecan, type II collagen or fibronectin. Moreover, the signalling pathways regulating these ADAMTS are poorly understood. Here we studied whether Runx2 and Wnt/β‐catenin, two signalling pathways involved in chondrocytes metabolism and OA pathology,^9^, ^10^, ^11^ are implicated in the production of ADAMTS‐7 and ‐12 in SF. Moreover, we analysed the role of interleukin‐1β (IL‐1β), and fibronectin fragments (Fn‐fs), two inflammatory mediators present in OA joints, as inducers of ADAMTS expression.^5^ Finally, we studied the involvement of ADAMTS‐7 and ‐12, and their signalling pathways in the regulation of COMP degradation in the cartilage‐SF interaction.

## 2 MATERIALS AND METHODS

### 2.1 Patients and synovial fibroblasts cultures

Synovial tissue was obtained from eight patients with active OA (five women and three men) aged between 65 and 87, at the time of total knee joint replacement surgery. Patients had advanced disease and were diagnosed of primary OA, excluding trauma, inflammatory disease and other structural causes of secondary OA. Control samples from healthy donors (HD) were obtained from five patients (three women and two men) aged between 50 and 72, at the time of knee arthroscopic evaluation. These patients were diagnosed with meniscopathy caused by trauma to the knee or sports injury, excluding inflammatory and rheumatic diseases. Samples were provided by the Rheumatology Service at Complejo Hospitalario Universitario A Coruña (CHUAC, A Coruña, Spain). The study was approved by the local Ethics Committee (Galicia, Spain) and performed according to the recommendations of the Declaration of Helsinki. All biopsy samples were obtained after subjects gave informed consent.

SF cultures were established by explant growth of synovial biopsies, cultured in Dulbecco's modified Eagle's medium (DMEM) with 25 mM HEPES and 4.5 g/L glucose, completed with 10% heat‐inactivated foetal bovine serum (FBS) (Lonza Ibérica SAU, Barcelona, Spain), 1% l‐glutamine and 1% antibiotic‐antimycotic (Invitrogen, Carlsbad, CA), at 37°C and 5% CO_2_. After three passages, residual contamination by macrophages was avoided, previously assessed by flow cytometry analysis of SF with a purity of 95%.^12^ Monocultures of SF were used for experiments until passage 6. Despite the use of cells at varying passage numbers, all comparisons within the same experimentation were made on SF at an identical passage number and at 80%‐90% confluent.

### 2.2 RNA extraction and RT‐qPCR

Synovial fibroblasts were seeded in 100‐mm petri dishes (3 × 10^5^ cells/dish) and cultured in serum‐free DMEM in the presence or absence of 10 ng/mL IL‐1β (ImmunoTools, Friesoythe, Germany), 10 nM 45 kDa Fn‐fs (Sigma‐Aldrich, St Louis, MO) or 100 ng/mL Wnt7B (Abnova, Taipei, Taiwan) for 24 hours. For inhibition experiments, cells were previously cultured in presence of 10 µM mitogen‐activated protein kinase (MAPK) p38 inhibitor (SB203580), 10 µM MAPK Kinase (MEK) and extracellular signal‐regulated kinase (ERK) inhibitor (PD98059) (Calbiochem, EMD Biosciences, San Diego, CA) or 200 ng/mL Dickkopf‐1 (DKK1) (R&D Systems, Abingdon, OX, UK) for 1 hour. Total RNA was obtained using Tri^®^Reagent (Sigma‐Aldrich). Two micrograms of RNA were reverse transcribed using a High Capacity cDNA Reverse Transcription Kit (Applied Biosystems, Foster City, CA). Semiquantitative real‐time PCR (qPCR) analysis was performed with a TaqMan® Gene Expression Master Mix with manufactured‐predesigned primers for ADAMTS‐7, ADAMTS‐12, Wnt3A, Wnt7B and β‐actin (Applied Biosystems). Assays were performed in triplicate. Results were normalized to β‐actin and plotted relative to the basal, using the ∆∆ cycle threshold method for quantification. In some cases, results were normalized to β‐actin and directly showed as mRNA expression (×10^4^).

### 2.3 Quantification of ADAMTS in culture supernatants

SF were seeded in 6‐well plates (6 × 10^4^ cells/well). Cells were cultured in serum‐free DMEM in the presence or absence of 10 ng/mL IL‐1β or 10 nM 45 kDa Fn‐fs for 24 hours. For inhibition experiments, cells were previously cultured in the presence of 10 µM SB203580, 10 µM PD98059 or 200 ng/mL DKK1 for 1 hour. Levels of ADAMTS were measured in the cell culture supernatants using commercial ELISA kits for ADAMTS‐7 and ADAMTS‐12 (MyBioSource, San Diego, CA).

### 2.4 Runx2 assay

Synovial fibroblasts were seeded in 150‐mm petri dishes (8 × 10^5^ cells/dish). Cells were cultured in serum‐free DMEM in the presence or absence of 10 ng/mL IL‐1β or 10 nM 45 kDa Fn‐fs for 60 and 30 minutes, respectively. Time‐course of Runx2 activation at 5, 15 and 30 minutes, and 1 and 2 hours of 10 ng/mL IL‐1β or 10 nM 45 kDa Fn‐fs treatment was previously performed. For inhibition experiments, cells were previously cultured in the presence of 10 µM SB203580 or PD98059 for 1 hour. A Nuclear Extract Kit (Active Motif, Rixensart, Belgium) was used for nuclear extracts preparation, and the protein content was measured with a QuantiProTM BCA Assay Kit (QBCA) (Sigma‐Aldrich). Nuclear extracts (12 µg/well) were added to a 96‐well plate and Runx2 activity was measured using a TransAM^TM^ AML‐3/Runx2 kit (Active Motif).

### 2.5 COMP assay in cartilage‐SF cultures

Release of COMP from OA cartilage was measured in the supernatants of SF cultures over cartilage explants.^13^ OA joint cartilages from four patients were provided by the Rheumatology Service at Complejo Hospitalario Universitario A Coruña (CHUAC, A Coruña, Spain). Fixed diameter (6 mm) sections were collected from adjacent areas to the injured cartilage. Samples were frozen at −80°C and stored until testing. One explant per well was attached to a 96‐well plate. HD‐ or OA‐SF were added drop‐wise on top of the cartilage surface (2 × 10^4^ SF per explant). After 3 hours of incubation, wells were filled with DMEM in the presence or absence of 10 µM PD98059 or 200 ng/mL DKK1 for 1 hour, followed by treatment with 10 ng/mL IL‐1β or 10 nM 45 kDa Fn‐fs. Cultures were continued for 7 days, and treatments were renewed every two days. Culture supernatants were collected for detection of COMP using a Quantikine® Human COMP Immunoassay (R&D Systems).

### 2.6 Statistical analysis

Statistical analysis was performed using GraphPad Prism software version 6 (GraphPad Inc, San Diego, CA). Data were subjected to normality test (Kolmogórov‐Smirnov test) and equal variance test (*F*‐test). Differences were assessed using Student's two‐tailed *t *test for two group comparisons, or one‐way analysis of variance (anova) for more than two groups, with a Bonferroni post hoc test for comparisons between each two groups. Results are presented as mean ± SEM. *P*‐values <0.05 were considered statistically significant.

## 3 RESULTS

### 3.1 ***Protein expression of ADAMTS‐7 and ***‐12*** in HD‐ and OA‐SF***

**Figure 1:**
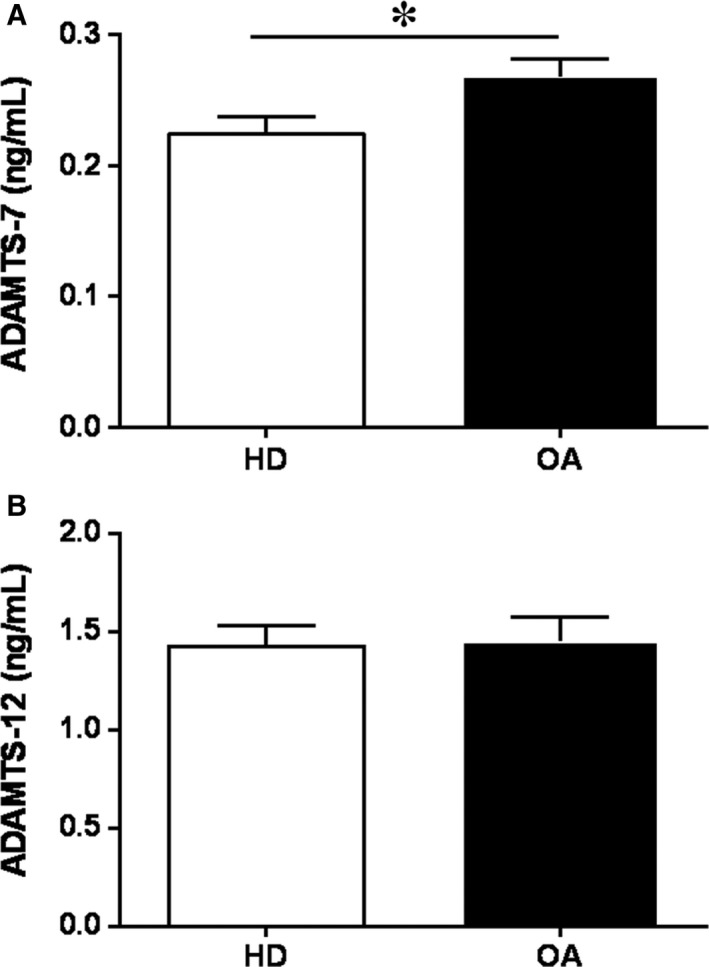
Constitutive protein expression of ADAMTS‐7 and ‐12 in HD‐ and OA‐SF. Protein secretion of ADAMTS‐7 (A) and ‐12 (B) was determined by ELISA in culture supernatants (n = 4 per experimental group). Data are presented as mean ± SEM of duplicate determinations. All comparisons with a p‐value less than 0.05 are shown. *P < 0.05 OA vs HD

To validate at protein level our previous results in ADAMTS‐7 and ‐12 mRNA transcripts,^5^ we performed ELISA analysis. Our data in culture supernatants confirmed that ADAMTS‐7 release was higher in OA than in HD‐SF (Figure 1A), while ADAMTS‐12 levels were similar in OA and HD‐SF (Figure 1B).

### 3.2 Runx2 and Wnt signalling in ADAMTS‐12 and ‐7 expressions

Runx2 and Wnt/β‐catenin pathways are involved in the ECM remodelling.^14^, ^15^, ^16^ As we previously reported the implication of the proinflammatory mediators IL‐1β and Fn‐fs in ADAMTS‐7 and ‐12 production,^5^ we now evaluated the involvement of Runx2 and Wnt/β‐catenin signalling in the expression of ADAMTS‐7 and ‐12 by SF stimulated with these two proinflammatory mediators. To this end, we treated the cells with PD98059 or SB203580 as inhibitors of ERK and p38 MAPK, respectively, two signalling pathways involved in Runx2 activation,^17^, ^18^ and with DKK1, a well‐known inhibitor of the canonical Wnt signalling.^19^, ^20^

#### 3.2.1 Runx2 is involved in ADAMTS‐12 expression

**Figure 2:**
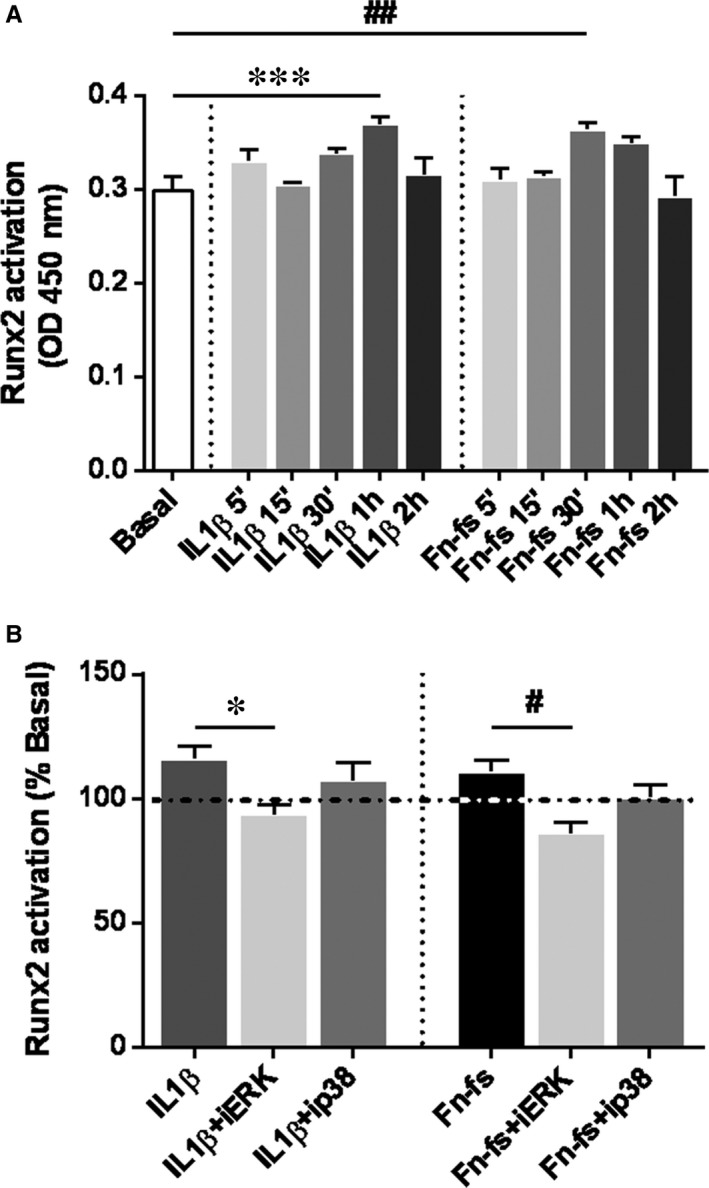
Activation of Runx2 in OA‐SF. A, Time‐course of Runx2 activation after 5, 15 and 30 minutes, and 1 and 2 hours of 10 ng/mL IL‐1β or 10 nM 45 kDa Fn‐fs treatment. B, Runx2 activation was measured in nuclear extracts by TransAM after 1 hour of treatment with inhibitors PD98059 (iERK) or SB203580 (ip38), followed by stimulation with IL‐1β for 60 minutes, or 45 kDa Fn‐fs for 30 minutes (n = 4 per experimental group). Data are presented as the percentage of activation relative to the basal (mean ± SEM of duplicate determinations). Dashed horizontal line represents the basal value. *P < 0.05, ***P < 0.001 vs IL‐1β, ^#^ *P* < 0.05, ^##^ *P* < 0.01 vs Fn‐fs

**Figure 3:**
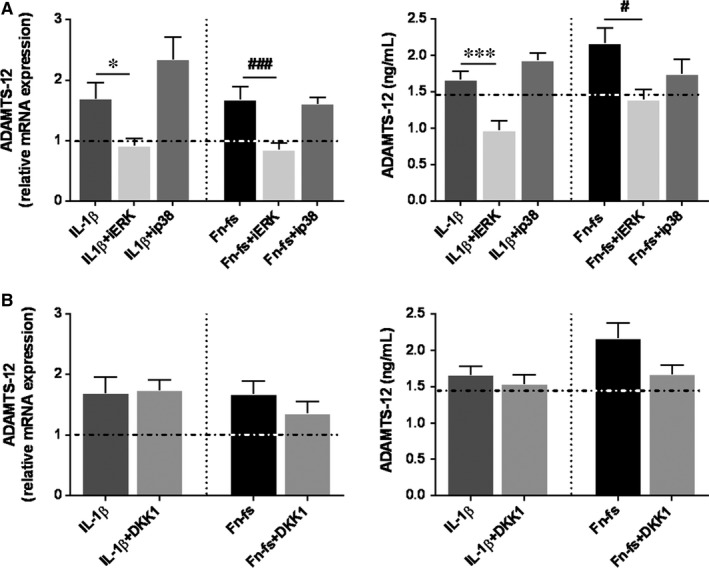
ADAMTS‐12 expression in OA‐SF after inhibition of Runx2 and Wnt/β‐catenin signalling. SF were treated with inhibitors PD98059 (iERK), SB203580 (ip38) or DKK1 for 1 hour, followed by treatment with IL‐1β or 45 kDa Fn‐fs for 24 hours (n = 4 per experimental group). (A, B) *left*. mRNA expression of ADAMTS‐12 was measured by RT‐qPCR. Data are presented as mean ± SEM of four independent samples analysed in triplicate (see Section 2) relative to the basal. (A, B) *right*. Secretion of ADAMTS‐12 was determined by ELISA in culture supernatants. Data are presented as mean ± SEM of duplicate determinations. Dashed horizontal lines represent the basal values. *P < 0.05, ***P < 0.001 vs IL‐1β; ^#^ *P* < 0.05, ^###^ *P* < 0.001 vs Fn‐fs

Time‐course of Runx2 showed increased activation at 60 and 30 minutes of 10 ng/mL IL‐1β and 10 nM 45 kDa Fn‐fs treatment, respectively (Figure 2A). To validate the participation of ERK and p38 MAPK in the induction of Runx2, we measured nuclear activation of Runx2 in cells pre‐treated with PD98059 or SB203580 and activated with IL‐1β or Fn‐fs at those time points. Our data showed that ERK inhibition decreased Runx2 activation in OA‐SF, triggered by IL‐1β or Fn‐fs stimulation (Figure 2B). By contrast, we detected no differences in cells isolated from HD (Figure S1). No effects were observed with the inhibitor of p38. Next, we studied whether ERK and p38 MAPK induction of Runx2, or Wnt signalling affected the expression and production of ADAMTS‐12. Only ERK inhibition decreased ADAMTS‐12 expression in OA‐SF, after IL‐1β or Fn‐fs stimulation (Figure 3A). No effects were observed with the inhibitor of p38, neither with the inhibitor of Wnt signalling, DKK1 (Figure 3B). No differences were observed when cells were obtained from HD (Figure S2). Together these data point to the involvement of ERK‐Runx2 axis in the expression of ADAMTS‐12 in OA‐SF.

#### 3.2.2 Wnt signalling is implicated in the ADAMTS‐7 expression

**Figure 4:**
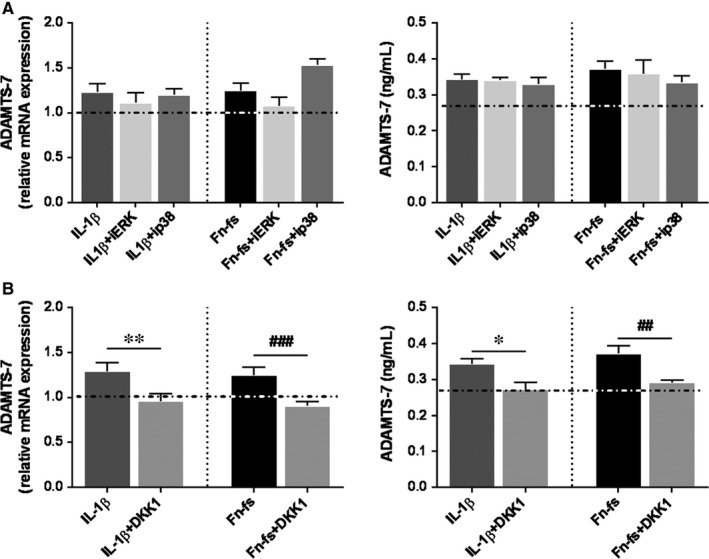
ADAMTS‐7 expression in OA‐SF after inhibition of Runx2 and Wnt/β‐catenin signalling. SF were treated with inhibitors PD98059 (iERK), SB203580 (ip38) or DKK1 for 1 hour, followed by treatment with IL‐1β or 45 kDa Fn‐fs for 24 hours (n = 4 per experimental group). (A, B) *left*. mRNA expression of ADAMTS‐7 was measured by RT‐qPCR. Data are presented as mean ± SEM of triplicate determinations (see Section 2) relative to the basal. (A, B) *right*. Secretion of ADAMTS‐7 was determined by ELISA in culture supernatants. Data are presented as mean ± SEM of duplicate determinations. Dashed horizontal lines represent the basal values. *P < 0.05, **P < 0.01 vs IL‐1β; ^##^ *P* < 0.01, ^###^ *P* < 0.001 vs Fn‐fs

**Figure 5:**
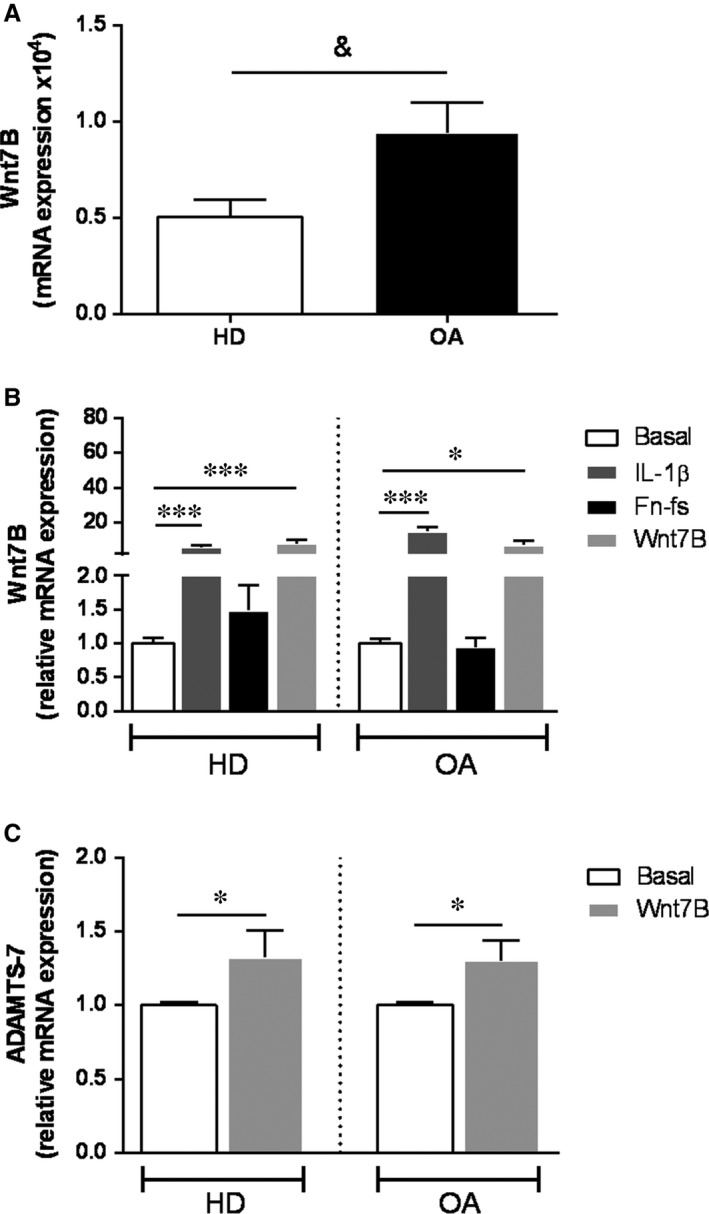
Expression of Wnt7B in HD‐ and OA‐SF. (A, B) mRNA expression of Wnt7B was measured by RT‐qPCR in HD‐ and OA‐SF (HD, n = 5 per experimental group; OA, n = 7 per experimental group). A, Constitutive mRNA expression of Wnt7B. B, mRNA expression of Wnt7B after treatment with IL‐1β, 45 kDa Fn‐fs or Wnt7B for 24 hours. C, mRNA expression of ADAMTS‐7 was measured by RT‐qPCR in HD‐ and OA‐SF after treatment with Wnt7B for 24 hours (n = 4 per experimental group). ^&^ *P* < 0.05 OA vs HD; *P < 0.05, ***P < 0.001 vs Basal. Values are presented as mean ± SEM of triplicate determinations (see Section 2)

DKK1 treatment decreased ADAMTS‐7 mRNA as well as protein levels in OA‐SF (Figure 4B), while only mRNA expression was reduced in HD (Figure S3B). We did not observe any effect in cells treated with MAPK inhibitors (Figure 4A, Figure S3A). Thus, these results suggest the involvement of Wnt/β‐catenin pathway in the expression of ADAMTS‐7 induced by IL‐1β or Fn‐fs in SF. We next studied whether these two pro‐inflammatory mediators modulated Wnt ligands expression. Firstly, we investigated whether SF expressed Wnt3A and Wnt7B, two members of the canonical Wnt signalling associated with OA physiopathology.^21^, ^22^ SF did not express Wnt3A (data not shown), while Wnt7B mRNA transcripts were detected in cells from HD and OA patients with a higher expression in OA‐SF (Figure 5A). Moreover, IL‐1β, but not Fn‐fs, induced Wnt7B expression (Figure 5B). In addition, Wnt7B also increased its own expression (Figure 5B), as well as ADAMTS‐7 mRNA transcripts in OA‐ as well as in HD‐SF (Figure 5C).

### 3.3 Blockage of COMP releasein cultures of OA‐SF over cartilage explants

**Figure 6:**
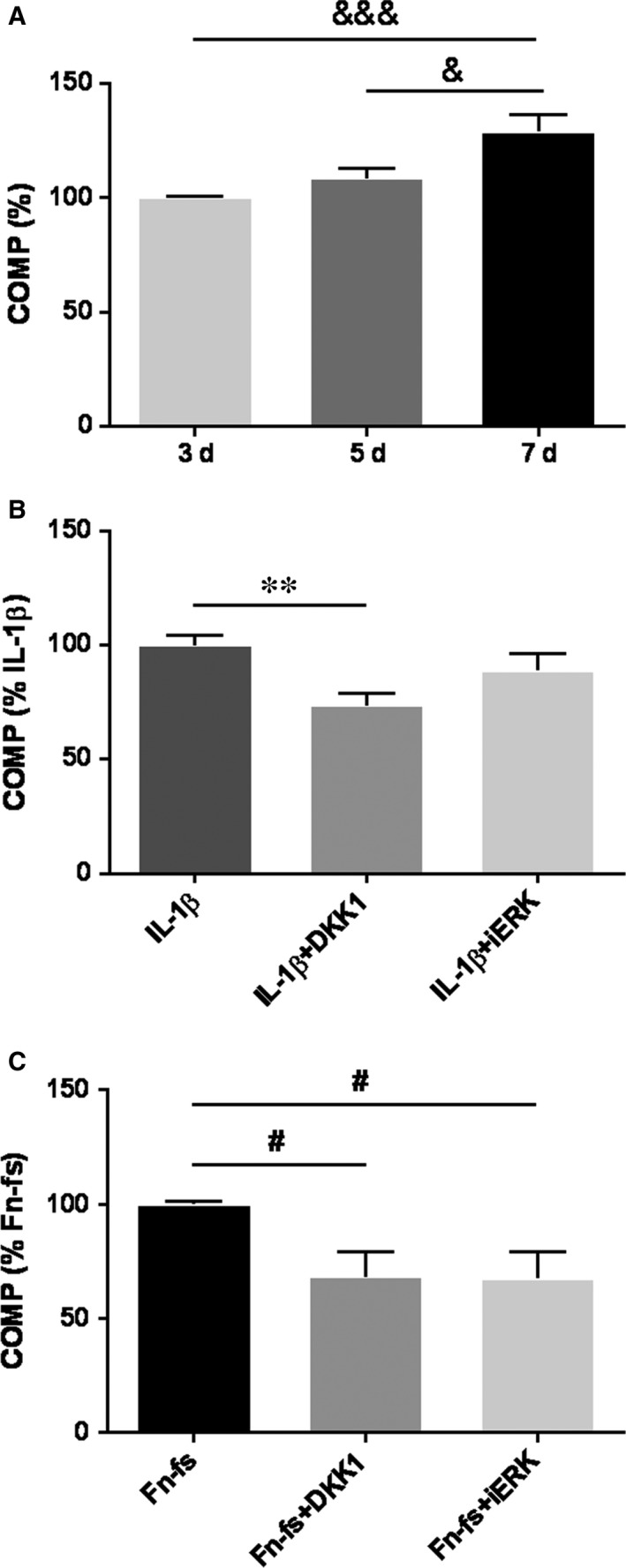
Blockage of COMP release in cultures of OA‐SF over cartilage explants. OA‐SF were seeded over cartilage explants and treated with IL‐1β or Fn‐fs for 7 days, with or without previous inhibition with DKK1 or PD98059 (iERK). COMP was detected by a Quantikine^®^ Human COMP Immunoassay in culture supernatants (n = 4 per experimental group). A, Time‐course of COMP at 3, 5 and 7 days at basal conditions. Data are presented as the percentage of 3 days (mean ± SEM of quadruplicate determinations). B, COMP concentration at day 7 of IL‐1β‐stimulation with DKK1 or iERK. Data are presented as the percentage relative to IL‐1β‐stimulation (mean ± SEM of quadruplicate determinations). C, COMP concentration at day 7 of 45 kDa Fn‐fs‐stimulation with DKK1 or iERK. Data are presented as the percentage relative to Fn‐fs‐stimulation (mean ± SEM of quadruplicate determinations). ^&^ *P* < 0.05, ^&&&^ *P* < 0.001 vs 7 days; **P < 0.01 vs IL‐1β, ^#^ *P* < 0.05 vs Fn‐fs

ADAMTS‐7 and ‐12 share a C‐terminal COMP/GEP‐binding domain, which is implicated in the degradation of COMP.^7^, ^8^ Thus, we studied the functional contribution of ADAMTS‐7 and ‐12 produced by SF in cartilage damage in OA. SF were cultured over healthy areas of cartilage explants from OA patients and COMP degradation was assessed in culture supernatants. We first determined the time course of COMP release from cartilage explants to the medium in the presence of OA‐SF, and observed the highest COMP release on day 7 compared to 3 and 5 days (Figure 6A). Cell cultures were also performed in presence of DKK1 or PD98059 inhibitors, followed by stimulation with IL‐1β or Fn‐fs. In cartilage explants co‐cultured with OA‐SF, the inhibition with DKK1 decreased the release of COMP triggered by both stimuli, while ERK signalling inhibition only decreased Fn‐fs‐induced COMP release (Figure 6B,C). Our data indicate that under inflammatory conditions (IL‐1β or Fn‐fs), OA‐SF trigger COMP degradation mainly via ADAMTS‐7, whereas ADAMTS‐12 is only active in presence of Fn‐fs. DKK1 treatment followed by stimulation with Fn‐fs was the only able to decrease COMP in the supernatants from cartilage explants cultured with HD‐SF (Figure S4).

## 4 DISCUSSION

In an inflammatory context, although OA is primarily characterized by chondrocytes degradation, other cells including SF also intervene through the activation of different biochemical pathways that trigger the breakdown of the cartilage ECM mediated by proteinases.^5^, ^23^ ADAMTS activity is essential in normal ECM remodelling processes. However, in OA dysregulation of metalloproteinases contributes to disease progression.^2^, ^4^ The role of collagenases such as MMP‐13, and aggrecanases (ADAMTS‐4 and ‐5) has been well documented.^2^, ^5^, ^24^ Emergent studies highlight the role of ADAMTS‐7 and ‐12 in inflammatory and rheumatic diseases, including RA and OA, atherosclerosis and cancer.^25^, ^26^, ^27^, ^28^ However, their function and signalling pathways involved in their expression, triggering the damage of non‐collagenous components of the ECM during OA, have not been well established yet. Here we report that protein release of ADAMTS‐7 in SF from OA patients is higher than in HD, whereas no differences were observed in ADAMTS‐12. These data agree with our previous findings on their mRNA levels.^5^

Osteoarthritis is a multifactorial pathology where many inflammatory and degradative mediators play essential roles. In this sense, IL‐1β is considered one of the main pleiotropic and proinflammatory cytokines in OA.^16^, ^29^ Besides, cartilage ECM degradation products are also relevant in the pathology.^30^ Amongst them, increased levels of fibronectin and its degradative fragments (Fn‐fs) have been described in cartilage and synovial fluid from OA and RA patients, also contributing to the degradative and inflammatory process favouring the chronicity of the disease.^31^, ^32^ NF‐kB and AP‐1 have been reported as master transcription factors in OA and many other inflammatory diseases, also implicated in the transcription of ADAMTS‐7.^28^, ^33^ However, little is known about other signalling pathways modulating the expression of ADAMTS‐7 and ‐12. Runx2 transcription factor and Wnt/β‐catenin signalling are involved in metalloproteinases gene expression and OA pathology, promoting the activation of catabolic mediators.^14^, ^15^, ^16^, ^34^, ^35^ We have previously described their role in the expression of ADAMTS‐4 and ‐5.^5^ Nonetheless, their association with ADAMTS‐7 and ‐12 is largely unexplored. Runx2 is a transcription factor involved in osteoblast differentiation, chondrocytes maturation and hypertrophy and matrix remodelling. It is up‐regulated in chondrocytes during OA,^16^, ^35^, ^36^ inducing the expression of MMP‐13 and ADAMTS‐5.^9^, ^14^, ^35^ In addition, two SNPs of Runx2 gene have been suggested as linked to OA.^35^, ^37^ Different signalling pathways are involved in the phosphorylation and activation of ADAMTS‐12.^17^ Amongst them, the MAPK especially ERK and p38, have been described as the main implicated in the modulation of Rux2‐mediated expression of ADAMTS‐5, and MMP‐13, in response to mechanical load or after IL‐1β stimulation with consequences in OA^9^, ^18^, ^38^, ^39^ Here we show that inhibition of ERK signalling decreases IL‐1β or Fn‐fs‐mediated activation of Runx2 in OA‐SF, and reduces ADAMTS‐12 expression. These data suggest the involvement of ERK‐Runx2 axis in the expression of this ADAMTS in OA‐SF. A previous report also demonstrated a fibroblast growth factor 2 (FGF2)‐mediated Runx2 induction of ADAMTS‐12 in OA cartilage.^34^

Wnt is a main regulator of joint remodelling, involved in cartilage and bone embryonic development.^15^, ^16^ Canonical Wnt pathway, which includes β‐catenin signalling, has also been widely implicated in OA pathogenesis.^11^, ^16^, ^22^, ^43^, ^44^ DKK1 is an upstream inhibitor of Wnt pathway,^46^ whose expression negatively correlates with OA progression.^36^ Here we report that DKK1 treatment reduces IL‐1β or Fn‐fs‐mediated ADAMTS‐7 expression, indicating an essential role of Wnt/β‐catenin signalling pathway in ADAMTS‐7 production in SF. Then we analysed the expression of two Frizzled receptor ligands of the canonical Wnt signalling implicated in the OA pathology. Wnt3A is a well‐known ligand, whose involvement in OA has been previously described in chondrocytes,^15^, ^22^, ^47^ Regarding Wnt7B, its up‐regulation has been described in OA and RA, with consequences in the pathology.^43^, ^48^ Wnt3A was no detected neither in SF from HD, nor in those from OA patients. Nevertheless, we show for the first time a higher expression of Wnt7B in OA‐SF compared to those from HD. In addition, IL‐1β up‐regulated the expression of Wnt7B, corroborating previous studies in chondrocytes,^19^ while no effects were observed after stimulation with Fn‐fs, which could be acting at another level along the Wnt/β‐catenin pathway or on other member of the Wnt family. Thus, we can conclude that the effect triggered by IL‐1β stimulation of Wnt//β‐catenin pathway in OA‐SF would be mediated by the induction of Wnt7B, which increases ADAMTS‐7 levels as well as its own expression in a positive feedback loop. Our results in these cells agree with other reports showing IL‐1β up‐regulation of Wnt//β‐catenin signalling through down‐regulation of Wnt antagonists in human chondrocytes.^49^ In addition to Wnt7B, the involvement of other members of the Wnt family in the OA pathology has also been described.^43^, ^48^ Moreover, further study would be needed as Runx2 and Wnt signalling mutually regulate their expression.^35^, ^50^, ^51^

COMP as a significant non‐collagenous component of cartilage ECM, plays an essential role in matrix assembly by the interaction with several ECM proteins.^53^ Increased levels of COMP and its degradative fragments have been detected in the serum and synovial fluid of OA and RA patients, and have been described as biomarkers of joint damage and disease progression.^2^, ^26^, ^53^, ^54^ Our results indicate a time‐course degradation of COMP from cartilage explants because of the action of OA‐SF, supporting the contribution of SF to COMP degradation. Moreover, in cartilage‐SF co‐cultures, Wnt//β‐catenin signalling blockade in presence of both, IL‐1β or Fn‐fs induces a decrease of COMP release, reflecting the increase in ADAMTS‐7 expression. Altogether these results point to a greater role for ADAMTS‐7 compared to ADAMTS‐12 in the degradation of COMP from cartilage ECM, mediated by SF in OA. However, inhibition of ERK‐Runx2 axis also reduced Fn‐fs‐mediated COMP degradation, suggesting the functional implication of ADAMTS‐12, which may act late in the course of the disease. All in all, both ADAMTS‐7 and ‐12 are involved in COMP degradation maintaining OA chronicity when Fn‐fs are present in the microenvironment. Our results are in agreement with previous reports describing that antibodies against ADAMTS‐7 and ‐12 additively inhibited COMP degradation in OA cartilage explants. Besides, interfering RNA silencing of these ADAMTS also prevented COMP degradation in chondrocytes from OA patients stimulated with IL‐1β or TNFα.^6^

**Figure 7:**
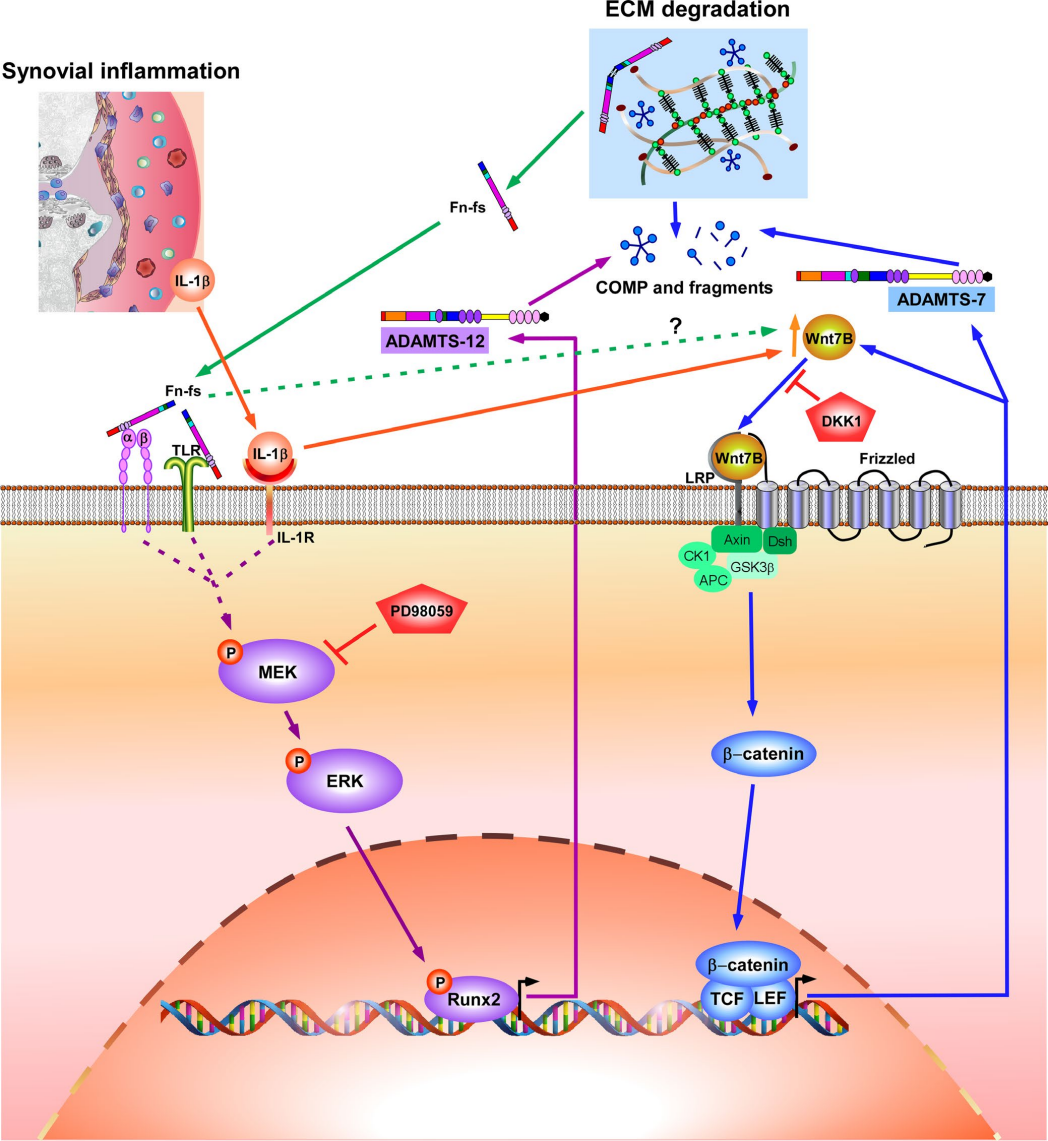
Schematic model of IL‐1β‐ and Fn‐fs‐mediated ADAMTS‐12 and ‐7 stimulation by Runx2 and Wnt/β‐catenin signalling modulation in OA‐SF. Synovial inflammation and cartilage ECM degradation are key events in OA pathology. SF and other cells in the joint produce proinflammatory mediators including IL‐1β. Fn‐fs are released to the microenvironment as consequence of ECM degradation. Binding of IL‐1β and Fn‐fs to their receptors, IL‐1 receptor (IL‐1R), and integrins (α, β) and Toll‐like receptors (TLRs), respectively, activates MEK‐ERK‐Runx2 axis. This activation converges in the transcription of ADAMTS‐12, with the subsequent degradation of COMP. Inhibition of MEK, and consequently ERK, with PD98059 (iERK), decreases Runx2 activation, ADAMTS‐12 transcription and COMP degradation from cartilage ECM. IL‐1β and Fn‐fs also activates Wnt/β‐catenin signalling, promoting β‐catenin binding to its nuclear cofactors TCF/LEF (T‐cell factor/lymphoid enhancer factor), and inducing ADAMTS‐7 expression, also converging in COMP cleavage from cartilage ECM. Activation of Wnt/β‐catenin by IL‐1β is mediated by induction of Wnt7B, which increases its own expression as well. Mechanisms involved in the stimulation of this signalling pathway by Fn‐fs are unknown. DKK1 inhibits this signalling pathway by blocking Wnt‐binding to its receptor, thus decreasing ADAMTS‐7 transcription and COMP degradation

The differences observed between OA‐ and HD‐SF should be taken into account to specifically target these ADAMTS under pathological conditions. While levels of ADAMTS‐7 are physiologically higher in OA‐ than in HD‐SF, similar modulation of its expression is observed in both. However, although OA‐SF‐mediated COMP degradation is inhibited by DKK1 with both stimuli, in HD‐SF this inhibition only occurs in the presence of Fn‐fs. Regarding ADAMTS‐12, despite its expression is similar in HD‐ and OA‐SF, when the stimuli are present, the blockade of ERK‐RUNX2 signalling and the subsequent COMP inhibition are only observed in OA patients. This specific modulation in OA‐SF supports the therapeutic value of these catabolic mediators. Collectively our data report for the first time, the involvement of ERK‐Runx2 axis and Wnt/β‐catenin signalling in the expression of ADAMTS‐12 and ADAMTS‐7 in SF, respectively, with the subsequent degradation of COMP from cartilage ECM (Figure 7).

Given the loss of cartilage regenerative capacity, the design of new therapies based on the blockade of mediators produced by neighbouring cells of the joint, such as SF, would help to prevent cartilage damage. In this sense, our results might contribute to the development of disease‐modifying OA drugs (DMOADs) focusing on the ADAMTS as targets for the prevention of ECM components degradation. In addition, the potential use of Wnt inhibitors as DMOADs is under study.^56^, ^57^ Nevertheless, given the involvement of ADAMTS‐7 and ‐12 in different stages of regeneration and destruction of cartilage and bone, further studies would be needed to design therapies based on the fine‐tuning of these signalling pathways.

## CONFLICT OF INTEREST

The authors confirm that there are no conflict of interest.

## AUTHORS’ CONTRIBUTIONS

SPG performed the research; RPG and YJ designed the research study; SPG, MC and RVR analysed the data; THG and MFM collected the samples; SPG and RPG wrote the paper; MM and FJB critically revised the paper.
