# Supplementary figures and images for: Wnt and RUNX2 mediate cartilage breakdown by osteoarthritis synovial fibroblast‐derived ADAMTS‐7 and ‐12
Source: J Cell Mol Med. 2019 Mar 22;23(6):3974–83. doi: 10.1111/jcmm.14283 (PMC6533528; doi:10.1111/jcmm.14283)

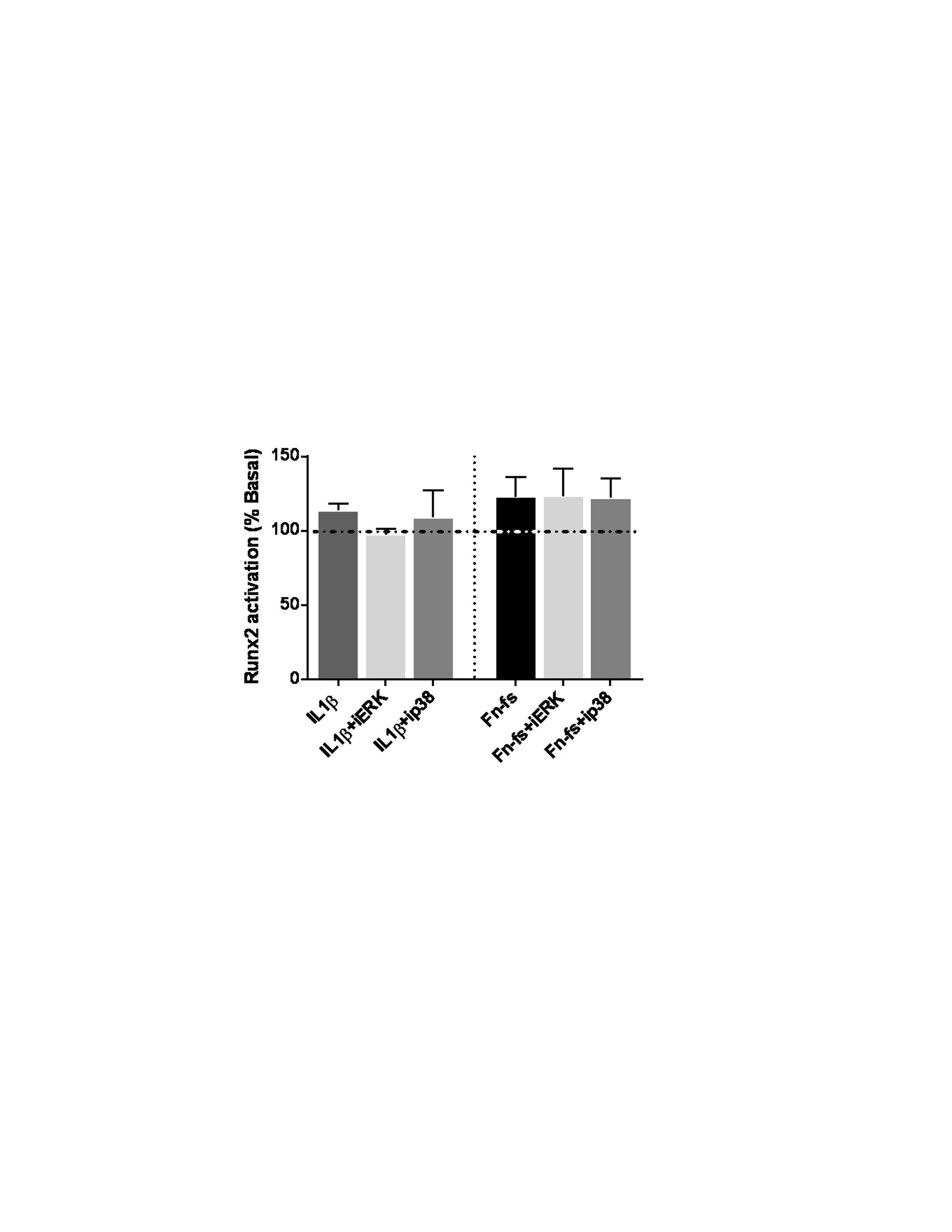

Supplement: Supplementary file 1 [file JCMM-23-3974-s001.tif]

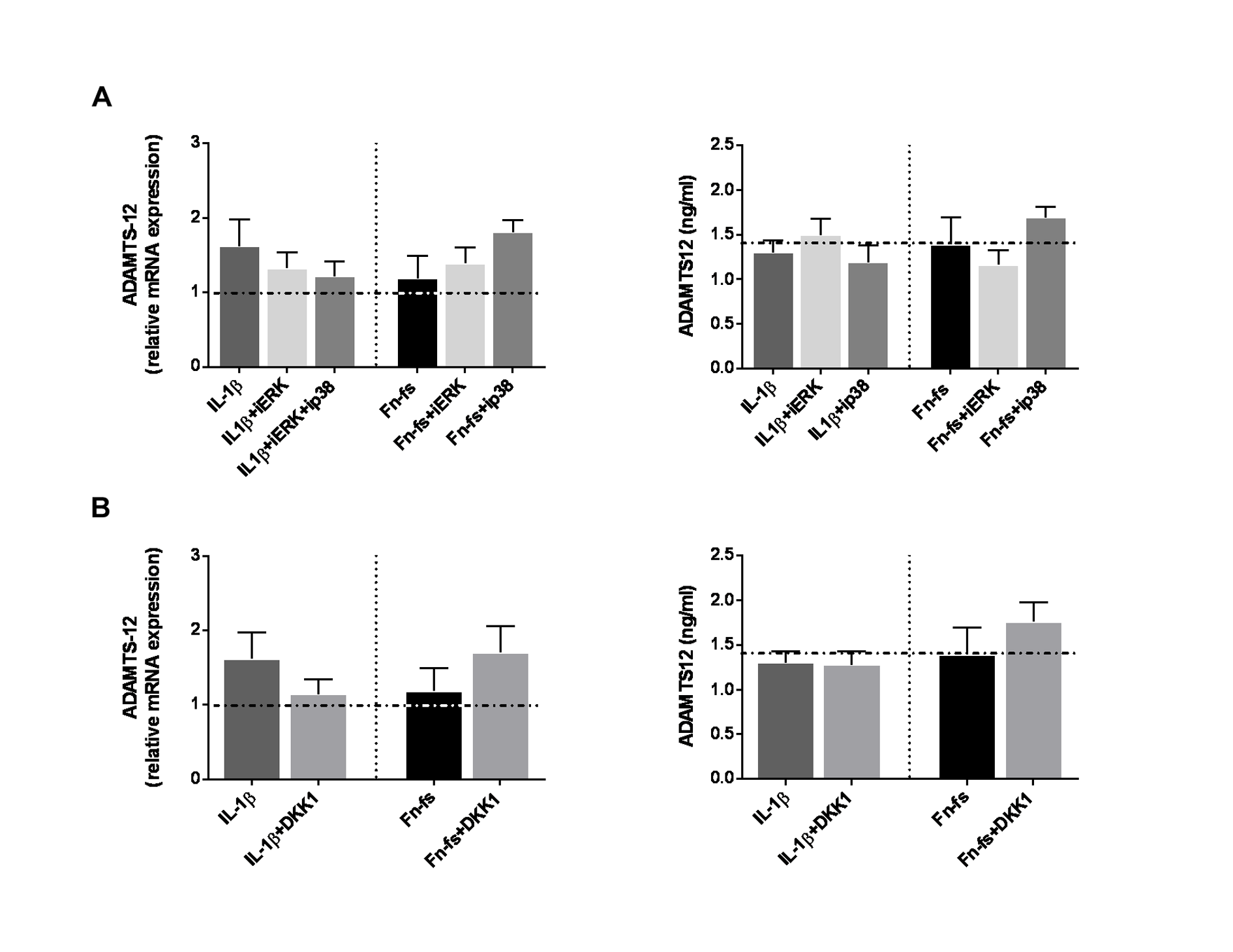

Supplement: Supplementary file 2 [file JCMM-23-3974-s002.tif]

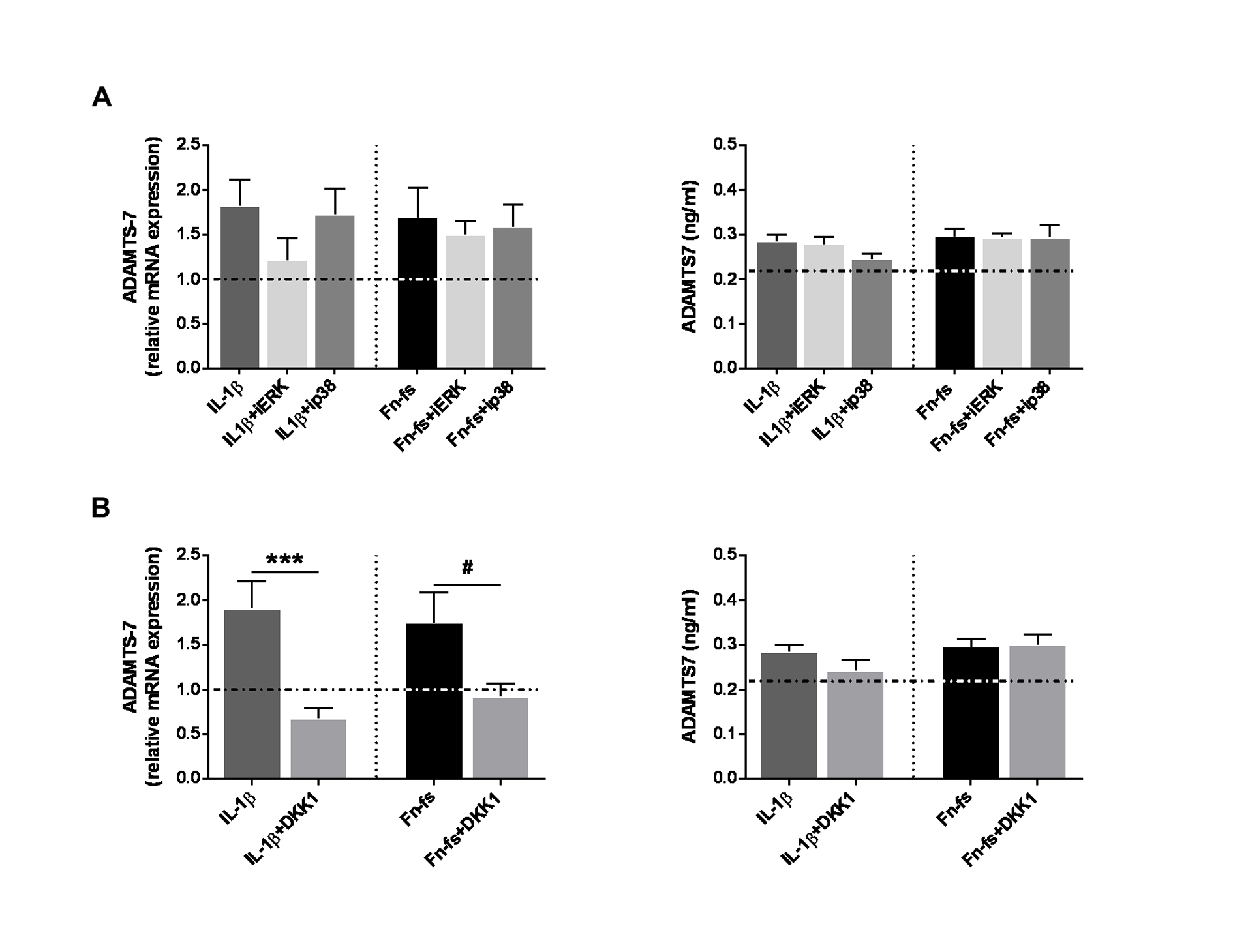

Supplement: Supplementary file 3 [file JCMM-23-3974-s003.tif]

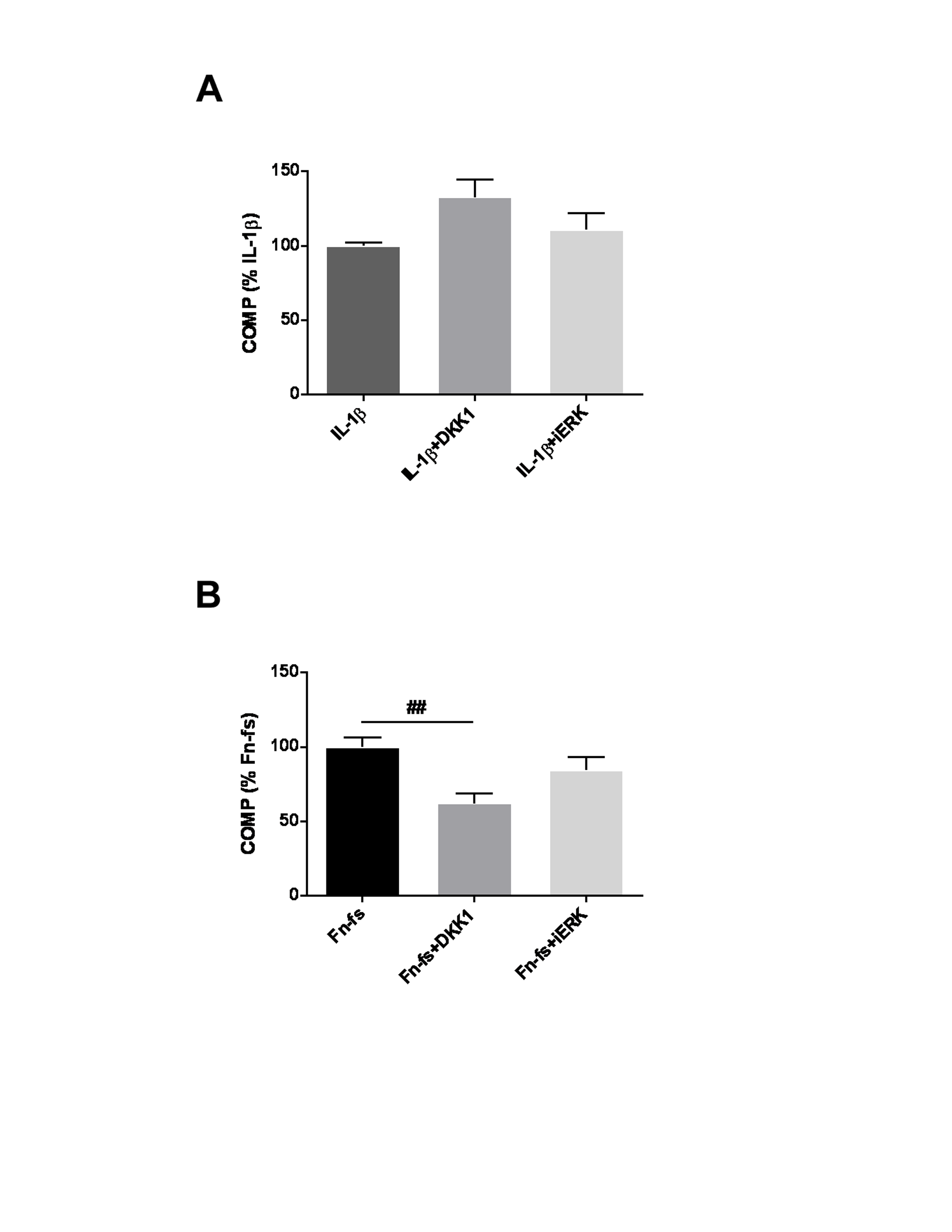

Supplement: Supplementary file 4 [file JCMM-23-3974-s004.tif]
